# Anastomotic Breakdown Five Years After Neoadjuvant Radiochemotherapy and Ultralow Anterior Resection for Rectal Adenocarcinoma

**DOI:** 10.7759/cureus.6861

**Published:** 2020-02-03

**Authors:** Eric Nguyen, Kristopher Dennis

**Affiliations:** 1 Radiation Oncology, Juravinski Cancer Centre, Hamilton, CAN; 2 Radiation Oncology, The University of Ottawa, Ottawa, CAN

**Keywords:** ultralow anterior resection, radiotherapy, chemotherapy, neoadjuvant, rectal cancer, stenosis, anastomotic dehiscence, breakdown, late toxicity, imaging

## Abstract

For carefully selected patients with low-lying rectal cancers, ultralow anterior resection (ULAR) can be an effective alternative to abdominal perineal resection, and together with neoadjuvant radiochemotherapy can provide the opportunity for sphincter preservation. However, ULAR is not without potential postoperative complications, particularly anastomotic dehiscence which increases in likelihood after receiving radiation therapy. While surveillance imaging is not indicated three years beyond initial surgical resection, changes in chronic symptoms refractory to conservative management may warrant further investigation. In this case report, we present an interesting case of late-onset stenosis and anastomotic breakdown following neoadjuvant radiochemotherapy, ULAR, and coloanal anastomosis for a low-lying rectal adenocarcinoma. Effective patient education, reliable symptom assessment, and multidisciplinary collaboration are essential to assessing for long-term treatment-related complications and providing appropriate treatment in a timely manner.

## Introduction

The goals of surgery for rectal cancer are cure, maintenance of function, and optimization of quality of life. For carefully selected patients with low-lying rectal cancers, ultralow anterior resection (ULAR) has become an alternative technique to abdominal perineal resection as it provides a chance for organ preservation [1]. When coupled with coloanal anastomosis (CAA), ULAR has been shown to allow normal continence and an acceptable frequency of bowel movements one year after surgery [1,2]. Neoadjuvant radiotherapy and chemotherapy have further increased the rate of sphincter preservation for patients with low rectal cancers [3].

Unfortunately, ULAR is associated with potential postoperative complications, the rates of which likely increase with the addition of radiation therapy [3]. Patients must be monitored for anastomotic dehiscence as this can cause bothersome symptoms, reduced anorectal function, abscess formation, and infection. Following curative intent multimodal therapy for locally advanced rectal cancers, patients typically enter a program of surveillance that includes endoscopic evaluation and imaging [4,5]. The goals of surveillance are to identify foci of recurrence and to monitor for and prevent complications. Local recurrence and significant complications typically present during the first few years following resection, and as such, imaging is seldom recommended to continue after five uneventful years on follow-up, and the interval between endoscopic evaluations is also typically lengthened to several years. However, for patients having undergone ULAR, careful attention must be paid to new symptoms even many years following treatment, as late events can still occur.

We report an interesting case of late-onset stenosis, anastomotic breakdown, and abscess formation following neoadjuvant radiation and chemotherapy, ULAR, and CAA for a low-lying rectal adenocarcinoma.

## Case presentation

A 67-year-old man with no comorbidities and no history of smoking was diagnosed with a cT2 N2 M0 rectal adenocarcinoma and was treated in 2008 with neoadjuvant pelvic radiotherapy (50.4 Gy/28 fractions), concurrent 5-fluorouracil-based chemotherapy, laparoscopic ULAR and CAA, and subsequent adjuvant chemotherapy with folinic acid, fluorouracil, and oxaliplatin. Radiotherapy consisted of a then standard four-field pelvic plan delivering 45 Gy/25 fractions to the primary tumor and mesorectal-, presacral-, and internal iliac lymph node regions followed by a sequential four-field boost plan to the primary tumor. Postoperative pathologic analysis revealed a well-differentiated rectal adenocarcinoma without evidence of perforation, perineural invasion, or lymphovascular invasion. The closest surgical margin was 1.5 cm from the distal edge, and the final pathologic stage was ypT2 ypN0.

The patient did well in follow-up over the next five years, without evidence of recurrent disease based on clinical assessments, colonoscopy, CT imaging, and carcinoembryonic antigen level monitoring. Unfortunately, he later developed fibrosis and stenosis within the anal canal, causing pain with defecation and pencil-thin stools. The anal canal and low rectum were appreciably narrowed and firm with marked restriction at the point of anastomosis 4 cm from the anal verge. His pain slowly increased in later years, presumed to be from worsening fibrosis. He eventually developed rectal frequency (upwards of 10 bowel movements per day), urgency, and occasional fecal incontinence. Initial medical intervention included oxycocet, proctosedyl suppositories, and topical creams containing 5% ketamine, 5% lidocaine, 6% gabapentin, and 2% baclofen. Despite these treatments, the patient continued to have severe pain peaking at 10/10 in intensity and bowel movements that had a significant impact on his quality of life. Dietary adjustments provided no improvement.

**Figure 1:**
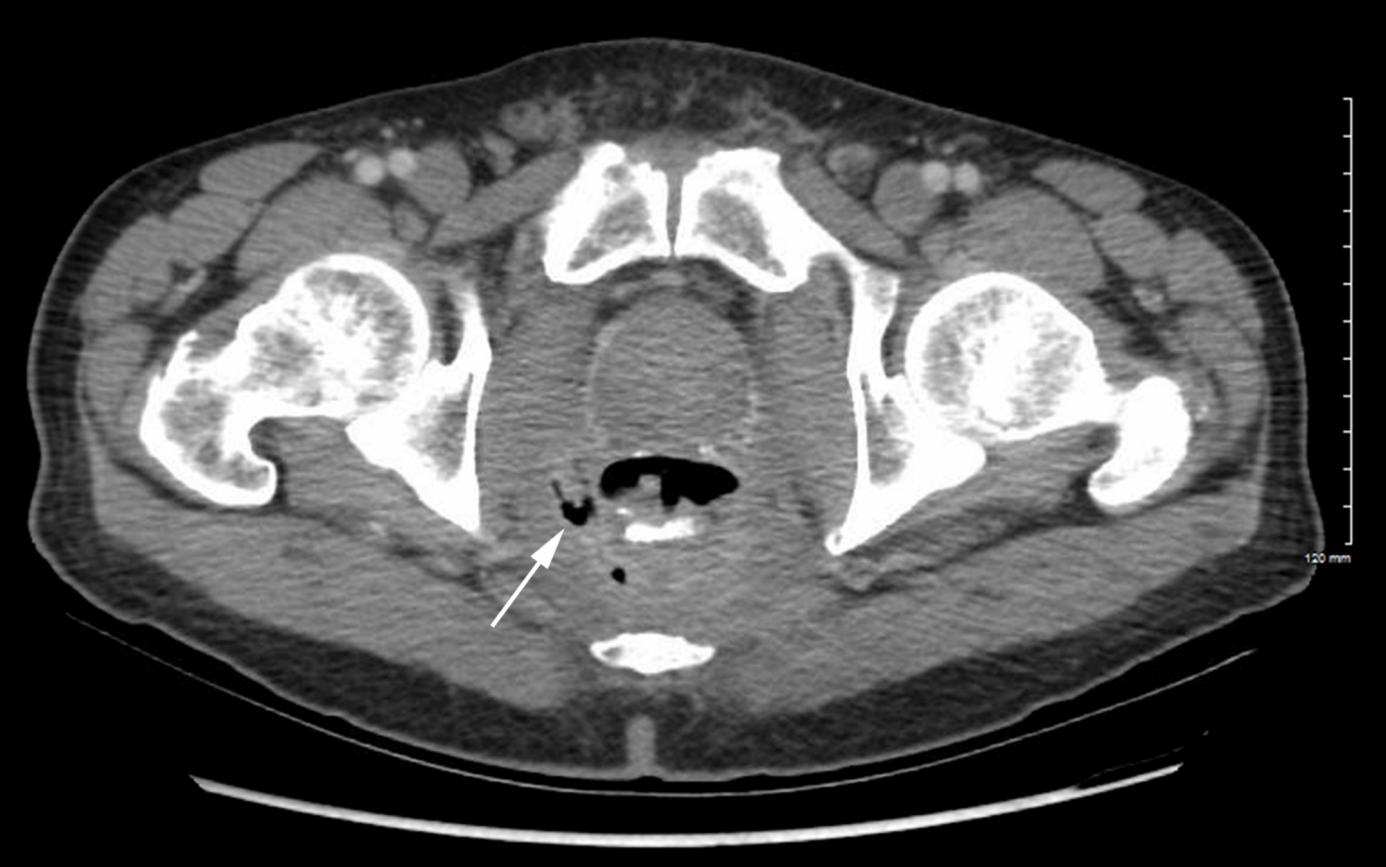
CT showing soft tissue and extraluminal air in the anastomosis

**Figure 2:**
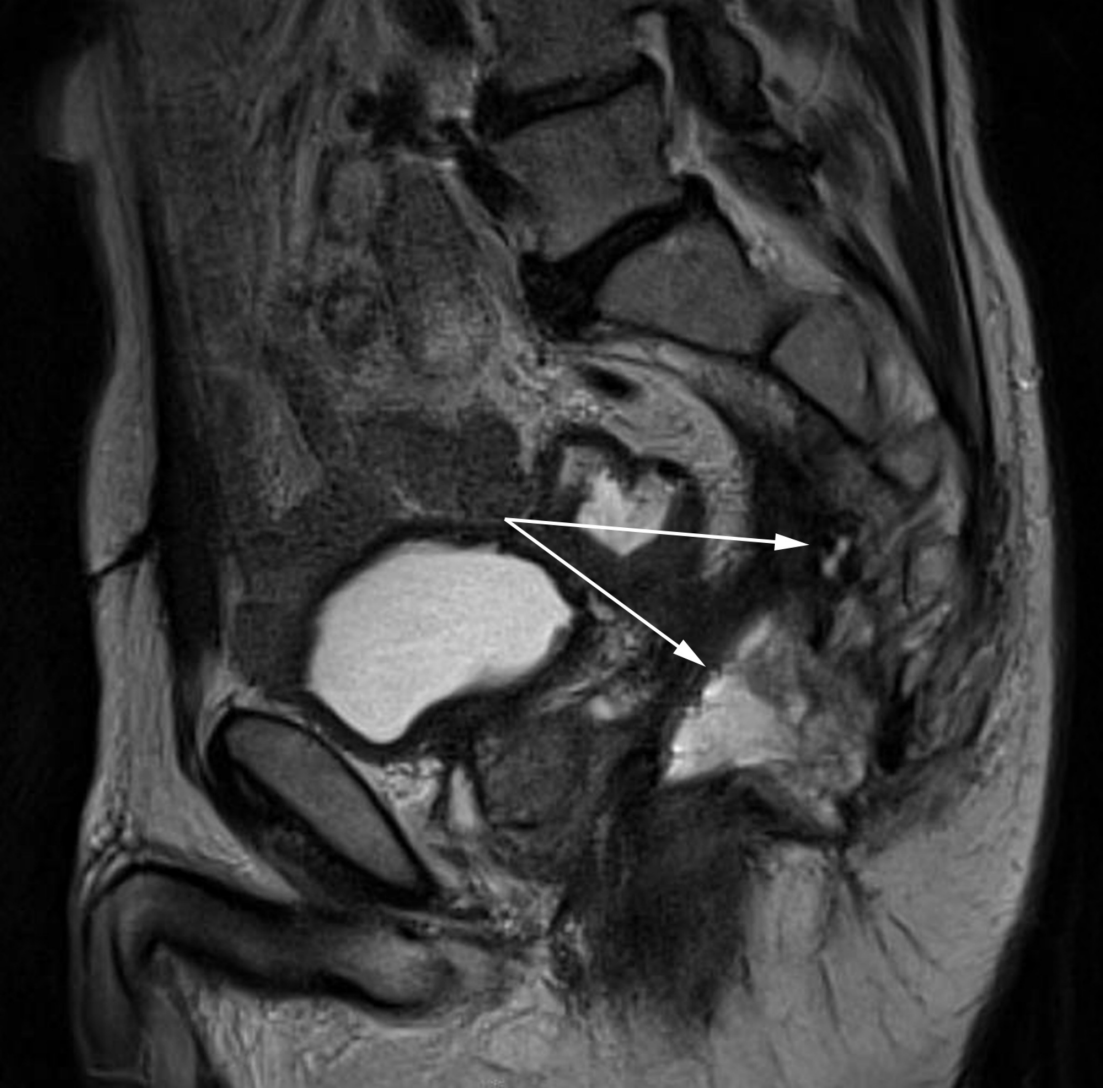
T2 MRI showing posterior wall rectal defect with a presacral abscess

A repeat CT revealed the presence of new soft tissue and extraluminal air proximal to the posterolateral aspect of the anastomosis (Figure 1). This represented anastomotic breakdown with contained perforation and perirectal inflammation. MRI confirmed a defect in the posterior wall of the rectum with communication to a presacral abscess (Figure 2). Biopsies of the soft tissue mass and whole body imaging were negative for local or distant recurrence. He was referred for surgical management and underwent abscess drainage with a permanent end colostomy in order to eliminate his pain and reduce his risk of subsequent infections. The patient did well postoperatively and was discharged with adequate pain control, and eventually tapered off of oral analgesics completely. He will continue to be followed with surveillance imaging and endoscopic evaluation.

## Discussion

The present case was an instance of low rectal cancer treated with multimodality therapy including ULAR and late-onset anastomotic dehiscence and abscess formation.

Preoperative radiotherapy in the treatment of rectal carcinomas can be beneficial among patients with a planned resection for locally advanced disease [6]. When used in conjunction with total mesorectal excision, radiotherapy has been found to reduce local recurrence, increase sphincter sparing, and reduce rates of positive margins [1,6,7]. However, radiation-induced damage to the anorectal area can lead to fibrosis, stenosis, and eventual bowel dysfunction [7,8]. It is estimated that 90% of patients develop a permanent change in their bowel habits after pelvic radiotherapy and surgery, 50% of which have an associated reduction in quality of life [9]. Patients can present with incontinence, rectal bleeding, mucoid discharge, tenesmus, abdominal cramps, and increased stool frequency [10,11].

Anastomotic dehiscence is a major cause of postoperative morbidity in patients with rectal cancer, with reported leakage rates from 3% to 20% after sphincter-saving resection [12]. The likelihood of anastomotic breakdown varies depending on the surgical method used, and the level of anastomosis has also been found to be an independent risk factor for leakage. The closer the anastomosis to the anal verge, the greater the chance of breakdown following surgery [13]. Some authors consider any level less than 6 cm above the anal verge as high risk for leakage [12,13]. Furthermore, it has been suggested that stapled anastomoses have better outcomes than hand-sewn anastomoses [13].

Radiotherapy has been identified as a risk factor for anastomotic leakage. Dehiscence of anastomoses was reported in 10% of patients following preoperative radiotherapy, with an increased risk in those receiving a short-course rather than long-course regimen [14]. This may be due to the persistent decrease in colorectal mural blood flow irrespective of the anastomotic method [15]. Increasing the time from the completion of short-course radiotherapy to surgery, however, has reduced complications [16].

While surgical technique and neoadjuvant therapies play a role in anastomotic breakdown, patient-related factors such as age greater than 60 years, male gender, preoperative medical disease, obesity, bowel obstruction, smoking, and alcohol abuse can significantly influence the probability of leakage [12,13]. Postoperative management should be catered to the patient based on risk assessment, and appropriate follow-up is essential regardless of efforts to reduce complications.

Cancer Care Ontario guidelines recommend surveillance imaging annually for three years following surgical resection for a colorectal malignancy [4]. Beyond this, it is unclear when to conduct follow-up imaging. For a patient well beyond surgery, CT and MRI are not indicated when patients are devoid of symptoms that would suggest recurrence. In the present case, there was no indication to image the patient based on his long-standing pain and thin stools due to stenosis. However, when his symptoms progressed in character, despite the time that had elapsed since surgery, a thorough investigation was imperative. Routine surveillance imaging may not be standard practice for patients at this stage following surgery, but it is worthwhile to investigate those with prior ULAR when their symptoms change.

## Conclusions

The present report serves as a reminder that maintaining an open line of communication between the patient and health care team is essential to identify possible recurrences or complications. The surgeon, radiation/medical oncologist, family physician, and pain and symptom specialists can all work together to devise an individualized treatment and surveillance plan.
